# Assessment of Colorectal Cancer Screening Disparities in U.S. Men and Women Using a Demographically Representative Sample

**DOI:** 10.1158/2767-9764.CRC-22-0079

**Published:** 2022-06-30

**Authors:** Sumit K. Shah, Marie-Rachelle Narcisse, Emily Hallgren, Holly C. Felix, Pearl A. McElfish

**Affiliations:** 1 Office of Community Health and Research, University of Arkansas for Medical Sciences Northwest, Fayetteville, Arkansas.; 2 College of Medicine, University of Arkansas for Medical Sciences Northwest, Fayetteville, Arkansas.; 3 Fay W. Boozman College of Public Health, University of Arkansas for Medical Sciences, Little Rock, Arkansas.

## Abstract

Timely receipt of colorectal cancer screening can reduce morbidity and mortality. This is the first known study to adopt Andersen's model of health services use to identify factors associated with colorectal cancer screening among U.S. adults. The data from National Health Interview Survey from 2019 was utilized to conduct the analyses. Multivariable logistic regression was used to separately analyze data from 7,503 age-eligible women and 6,486 age-eligible men. We found similar colorectal cancer screening levels among men (57.7%) and women (57.6%). Factors associated with higher screening odds in women were older age, married/cohabitating with a partner, Black race, >bachelor's degree, having a usual source of care, and personal cancer history. Factors associated with lower odds for women were American Indian/Alaska Native race, living in the United States for ≤10 years, ≤138% federal poverty level (FPL), uninsured or having Medicare, and in fair/poor health. For men, factors associated with higher screening odds were older age, homosexuality, married/cohabitating with a partner, Black race, >high school/general educational development education, having military insurance, having a usual source of care, and personal cancer history. Factors associated with lower odds for men were being a foreign-born U.S. resident, living in the South or Midwest, ≤138% FPL, and being uninsured or having other insurance. Despite lower screening rates in the past, Black adults show a significantly higher likelihood of colorectal cancer screening than White adults; yet, screening disparities remain in certain other groups. Colorectal cancer screening efforts should continue to target groups with lower screening rates to eliminate screening disparities.

**Significance:**

Timely receipt of colorectal cancer screening can reduce morbidity and mortality. Identification of populations and domains of factors associated with colorectal cancer screening receipt among men and women can help future interventions to alleviate impeding factors and target screening promotion efforts in populations not adherent with screening guidelines.

## Introduction

Cancer screenings help identify cancers early and mitigate cancer-associated morbidity and mortality and the growing financial burden of cancer treatment (1). Colorectal cancer is the second most common cancer in men and women in the United States (2). Screening for colorectal cancer can detect precancerous polyps and cancerous lesions in early stages which can be treated effectively (1). As of 2019, the U.S. Preventive Services Task Force (USPSTF) recommended adults aged 50 to 75 years should screen for colorectal cancer using one of a variety of screening options. Research has shown a 60% reduction in mortality and a 73% increase in the 5-year survival rate for adults adherent with colorectal cancer screening recommendations (3). However, more than 60% of colorectal cancer cases are diagnosed when they have either spread locally or metastasized to distant organs due to delayed detection (2).

The Healthy People 2030 goal for colorectal cancer screening among age-eligible adults is 74.4% (4). However, the 2018 National Health Interview Survey (NHIS) revealed only 66.9% of age-eligible adults were screened, with rates similar for men (67.4%) and women (66.5%; ref. 5). Research has identified factors associated with not having colorectal cancer screenings, including lack of awareness, screening test knowledge, social support, access to health care services, and insurance coverage; negative attitudes and beliefs; lower education and income; and language barriers for foreign-born residents (6, 7). However, only a few colorectal cancer screening studies have used nationally-representative data to examine colorectal cancer screening (5, 8, 9), and even fewer studies have rarely identified determinants of screening uptake for men and women separately. No studies were identified that applied the comprehensive Andersen's model of health services (Andersen's model) use using a national representative sample. This study was undertaken to fill these gaps in the literature and to contribute to scientific knowledge of colorectal cancer screening by examining variations in screening receipt using a nationally representative dataset, the NHIS (10).

The Andersen's model categorizes factors affecting service use into three domains: “predisposing,” “enabling,” and “need” factors (11). Predisposing factors are sociodemographic characteristics (e.g., age, sex, race/ethnicity, education level, country of birth) related to service use. Enabling factors are resources that facilitate service use if they are available (e.g., health insurance, transportation). Need factors are real and perceived health conditions that may warrant service use (e.g., medical history, health status; refs. 11, 12). Identification of individual and domains of factors associated with colorectal cancer screening among men and women can inform interventions to target those who are not adherent with screening guidelines.

## Materials and Methods

### Data Source

The study used data from the 2019 NHIS. The NHIS is conducted annually by the National Center for Health Statistics to monitor the health of the U.S. population on a broad range of health topics by surveying a representative random sample of the U.S. civilian noninstitutionalized population. Data are collected through personally interviewing one adult from each household randomly selected to answer detailed questions about their demographic information and health. More information about the NHIS can be found elsewhere (13).

### Study Population

There were 15,989 respondents to the 2019 NHIS who were age-eligible (50–75 years) for USPSTF colorectal cancer screening. As the focus of the study was on routine colorectal cancer screening, respondents who reported colorectal cancer screening for reasons other than a routine examination were excluded from the study population (*n* = 1,876), resulting in a final analytic sample of 13,989 (women = 7,503, men = 6,486).

### Measures

#### Outcome

Being up-to-date with colorectal cancer screening was defined as receiving a recommended screening test at specified intervals as per the USPSTF guidelines (see Table 1). The tests recommended are: High-sensitivity guaiac fecal occult blood test (gFOBT), fecal immunochemical test (FIT), stool DNA test with FIT (sDNA-FIT), CT/virtual colonography, flexible sigmoidoscopy, flexible sigmoidoscopy with FIT, and colonoscopy. The NHIS questionnaire asked respondents to indicate whether they had received any of those tests, the time since they last received the test, and whether the screening was part of a routine examination. Respondents were coded as having had a recommended colorectal cancer screening if they had had one of those recommended screening tests within the recommended frequency for the test as part of a routine examination. Respondents were coded as not having a recommended colorectal cancer screening if they had not had one of the tests or had had one test but outside the USPSTF recommended frequency. Response options “Refused,” “Not ascertained,” and “Don't know” were coded as missing.

**TABLE 1:** USPSTF Colorectal Cancer Screening Guidelines

Age range	Screening tests	Screening frequency
Adults between the ages of 50–75 years	High-sensitivity guaiac fecal occult blood test (gFOBT)	Every year
	Fecal immunochemical test (FIT)	
	Stool DNA test with FIT (sDNA-FIT)	Every 1–3 year(s)
	CT colonography	Every 5 years
	Flexible sigmoidoscopy	
	Flexible sigmoidoscopy with FIT	Flexible sigmoidoscopy every 10 years plus FIT every year
	Colonoscopy	Every 10 years

NOTE: Source: U.S. Preventive Service Task Force, colorectal cancer screening recommendations (2016).

Factors representing the Andersen's model three domains were selected for the analysis based on previous research (14, 15). The operational definitions of the variables are shown in Supplementary Table S1.

#### Predisposing Factors

Age, sexual orientation, race/ethnicity, education, marital status, nativity, urban-rural residence classification, and region of residence.

#### Enabling Factors

Employment status in past 12 months, income level, health insurance coverage, problems paying medical bills in past 12 months, worry about paying medical bills if sick/in an accident, usual source of medical care, and number of children in household.

#### Needs Factors

Perceived health status, personal history of cancer, and body mass index (BMI) categories.

### Statistical Analysis

We computed weighted percentages and weighted 95% confidence intervals (CI) for categorical variables and weighted means and SEs for continuous variables to describe age-eligible adults by sex who were up-to-date with USPSTF colorectal cancer screening.

In separate multivariable logistic regression models for men and women, we estimated the association between colorectal cancer screening and the selected predisposing, enabling, and need factors. The magnitude and direction of the associations were captured by odds ratios (ORs), and the uncertainty around the estimates were captured by 95% CIs.

Multicollinearity among independent variables was assessed by computing the variance inflation factor. Statistical significance was determined at an *a priori* α = 0.05. The complex design of the survey was accounted for with sampling adult weights and other design variables. All descriptive and regression analyses were conducted with STATA/SE 16 (16).

### Data Availability

The data analyzed in this study were obtained from the National Center for Health Statistics NHIS at https://www.cdc.gov/nchs/nhis/2019nhis.htm.

## Results

### Colorectal Cancer Screening

Within the study population, 57.6% of women and 57.7% of men received routine colorectal cancer screening as recommended by USPSTF guidelines.

### Characteristics of Age-Eligible Women and Men Who Received a Colorectal Cancer Screening

Results of the analyses to profile women and men up-to-date with colorectal cancer screening based on the predisposing, enabling, and need factors are described below and also in Supplementary Table S1.

#### Women

##### Predisposing Factors

The mean age of women up-to-date with colorectal cancer screening was 62.9 years (SE:0.11). More than half (58.4%) of straight women and 51.7% of homosexual women received colorectal cancer screening. Receipt of screening was lowest among American Indian/Alaska Native (AI/AN) women (36.2%), followed by Hispanic (46.5%), Asian (51.7%), other race/ethnicity (55.7%), White (59.8%), and Black (60.2%) women. Women with less than high school/general educational development education (HS/GED) had the lowest colorectal cancer screening (46.8%) versus women with more education. More than half of (59.7%) women born in the United States were up-to-date with colorectal cancer screening versus 24.3% of foreign-born women living in the United States for ≤10 years. Women living in the Midwest had the highest percentage of colorectal cancer screening (60.3%). Women living in the South had the lowest percentage of colorectal cancer screening (54.9%). The distribution of colorectal cancer screening in large central, large fringe, and medium-small metropolitan areas was 58.0%, 57.5%, and 58.7%, respectively.

##### Enabling Factors

Over half of employed women (54.5%) received colorectal cancer screening vs. 61.6% of unemployed women. Women who had problems paying medical bills (50.4%) had lower screening uptake than women without problems paying medical bills (59.2%). Only 47.0% of women who were very worried about medical bills received the screening versus 63.0% of women who were not at all worried about it. Less than half (44.6%) of less affluent women (incomes ≤138% FPL) received colorectal cancer screening vs. 64.5% of women with incomes >400% FPL. Uninsured women had the lowest percentage of receiving colorectal cancer screening (28.2%). For the other health insurances, the percentages were: Medicaid 46.6%; military insurance, 56.5%; Medicare, 61.2%; and private insurance, 60.2%. Twenty-seven percent of women without a usual source of medical care received colorectal cancer screening versus 60.3% with usual source of care in a doctor's office and 43.6% with usual source of care in other medical facilities. The mean number of children in the household was 0.17 (SE:0.01) for women up-to-date with colorectal cancer screening.

##### Need Factors

Among women in excellent health, 61.5% had received colorectal cancer screening versus 50.5% of women in fair/poor health. Colorectal cancer screening levels were nearly the same for women who were overweight or obese (57.5% and 57.9%). Among women diagnosed with cancer, 66.2% were up-to-date with colorectal cancer screening versus 56.2% without a history of cancer.

#### Men

##### Predisposing Factors

The mean age of men up-to-date with colorectal cancer screening was 62.6 (SE:0.13). More than half (58.3%) of straight men and 71.8% of homosexual men received colorectal cancer screening. Six in ten (61.7%) men who were married/cohabitating received colorectal cancer screening vs. 49.1% of unmarried men. Less than half of Hispanic men (41.8%) were up-to-date with colorectal cancer screening, followed by Asian (47.0%), AI/AN (47.3%), other race/ethnicity (51.8%), Black (58.3%), and White (61.3%) men. Men with <HS/GED were least up-to-date with colorectal cancer screening (41.1%). Six in 10 men (61.4%) who were born in the United States were up-to-date with colorectal cancer screening vs. 46.6% of foreign-born men who had resided in the United States >10 years and 19.2% of foreign-born men living in the United States for ≤10 years. About 63.6% of men living in the Northeast, 56.7% living in the Midwest, and 56.7% living in the West were up-to-date with colorectal cancer screening. Men living in the South had the lowest screening uptake (55.6%).

The distribution of colorectal cancer screening in large central, large fringe, and medium-small metropolitan areas was 54.2%, 62.4%, and 59.4%, respectively.

##### Enabling Factors

More than half of employed men and unemployed men (55.2% and 62.7%) received colorectal cancer screening. About 48.3% of men with problems paying medical bills received the screening versus 59.3% of those without such financial problems paying medical bills. Among men with incomes ≤138% FPL, 40.1% received colorectal cancer screening versus 66.9% of men with incomes >400% FPL. Uninsured men were least up-to-date with colorectal cancer screening (21.2%), followed by men with Medicaid (41.8%), other insurance (60.1%), private insurance (61.4%), Medicare (63.0%), and military insurance (67.7%). Only 18.3% of men without a usual source of care received colorectal cancer screening versus 62.1% with a usual source of care at a doctor's office and 54.5% who had other places as a usual source of care. The mean number of children in the household for men who received colorectal cancer screening (57.7%) was 0.19 (SE:0.01).

##### Need Factors

The highest proportion of colorectal cancer screening was reported among men in excellent and very good overall health (61.8% and 60.9%, respectively) versus 52.5% of men in fair/poor overall health and 55.8% of men in good health. Colorectal cancer screenings among overweight and obese men were similar (58.7% and 58.3%, respectively). Among men diagnosed with cancer, 71.4% received colorectal cancer screening versus 55.5% who were not diagnosed with cancer.

### Adjusted Associations Between Predisposing, Enabling, and Need Factors and Colorectal Cancer Screening Among Age-Eligible Women and Men

**FIGURE 1:**
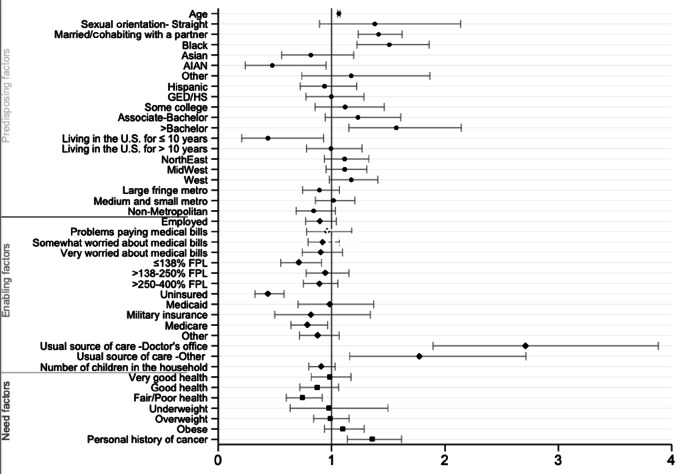
Predicting factors associated with receipt of colorectal cancer screening in women. Multivariable logistic regression results showing ORs with CI values of various predisposing, enabling, and need factors and their associations with colorectal cancer screening uptake in women.

**FIGURE 2:**
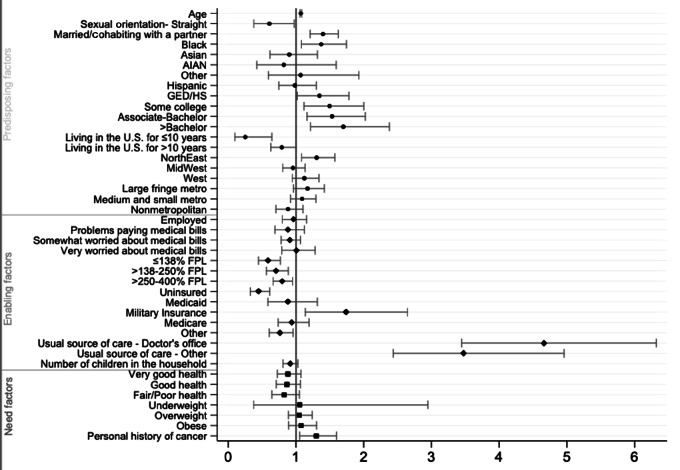
Predicting factors associated with receipt of colorectal cancer screening in men. Multivariable logistic regression results showing ORs with CI values of various predisposing, enabling, and need factors and their associations with colorectal cancer screening uptake in men.

Adjusted associations between predisposing, enabling, and need factors and colorectal cancer screening from sex-specific multivariable logistic regression analyses are reported below, in Table 2, and in Figures 1 and 2.

**TABLE 2:** Characteristics associated with colorectal cancer screening among women and men

	Women		Men	
Variables	OR (95% CI)^a^		OR (95% CI)^a^	
**Predisposing factors**				
**Age**	**1.06^b^ (1.05–1.08)**		**1.07^b^ (1.06–1.09)**	
Sexual orientation				
Homosexual	Ref	—	Ref	—
Straight/heterosexual	1.38	0.89–2.14	0.61^c^	0.38–0.98
Marital status				
Unmarried	Ref	—	Ref	—
Married/cohabiting with a partner	1.41^b^	1.23–1.62	1.40^b^	1.21–1.62
Race/Ethnicity				
White	Ref	—	Ref	—
Black	1.51^b^	1.22–1.86	1.37^c^	1.08–1.74
Asian	0.82	0.56–1.20	0.90	0.61–1.32
AI/AN	0.48^c^	0.24–0.95	0.82	0.42–1.59
Hispanic	0.94	0.72–1.22	1.07	0.59–1.93
Other race/ethnicity	1.17	0.74–1.87	0.98	0.75–1.30
Educational attainment				
<HS/GED	Ref	—	Ref	—
HS/GED	1.00	0.77–1.29	1.35^c^	1.02–1.78
Some college/no degree	1.12	0.86–1.46	1.50^c^	1.12–2.00
Associate-Bachelor's	1.23	0.94–1.61	1.53^d^	1.16–2.02
>Bachelor's	1.57^d^	1.15–2.14	1.70^d^	1.21–2.38
Nativity				
U.S. citizen by birth	Ref	—	Ref	—
Living in the United States for ≤10 years	0.44^c^	0.21–0.93	0.25^d^	0.10–0.64
Living in the United States for >10 years	0.99	0.78–1.27	0.79^c^	0.63–1.00
Region of residence				
Northeast	Ref	—	Ref	—
Midwest	1.00	0.82–1.22	0.73^d^	0.59–0.90
South	0.90	0.75–1.07	0.77^d^	0.64–0.92
West	1.05	0.86–1.29	0.86	0.70–1.11
Area of residence				
Large central metropolitan	Ref	—	Ref	—
Large fringe metropolitan	0.89	0.75–1.07	1.17	0.96–1.42
Medium-small metropolitan	1.02	0.86–1.20	1.09	0.92–1.29
Non-metropolitan	0.84	0.69–1.03	0.88	0.70–1.11
**Enabling factors**				
Employment status				
Unemployed	Ref	—	Ref	—
Employed	0.90	0.77–1.04	0.96	0.80–1.16
Problems paying medical bills				
No problems paying medical bills	Ref	—	Ref	—
Problems paying medical bills	0.96	0.78–1.18	0.88	0.69–1.12
Worry about paying medical bills				
Not at all worried	Ref	—	Ref	—
Somewhat worried	0.92	0.79–1.07	0.91	0.78–1.06
Very worried	0.90	0.74–1.10	1.01	0.79–1.28
Federal poverty level				
>400% FPL	Ref	—	Ref	—
≤138% FPL	0.71^c^	0.55–0.91	0.59^b^	0.45–0.77
>138%–250% FPL	0.95	0.78–1.15	0.71^d^	0.56–0.89
>250%–400% FPL	0.89	0.75–1.05	0.79^c^	0.66–0.95
Insurance coverage				
Private insurance	Ref	—	Ref	—
Uninsured	0.43^b^	0.33–0.58	0.45^b^	0.33–0.61
Medicaid	0.98	0.70–1.37	0.88	0.59–1.32
Military insurance	0.82	0.50–1.34	1.74^c^	1.14–2.65
Medicare	0.79^c^	0.64–0.96	0.94	0.74–1.19
Other insurance	0.87	0.72–1.07	0.76^c^	0.60–0.96
Usual source of care				
No usual source of care	Ref	—	Ref	—
Usual source of care–Doctor's office	2.71^b^	1.89–3.88	4.67^b^	3.45–6.32
Usual source of care–Other medical facility	1.77^d^	1.16–2.71	3.48^b^	2.44–4.96
Children in household	0.91	0.80–1.03	0.91	0.81–1.03
**Need factors**				
Health status				
Excellent health	Ref	—	Ref	—
Very good health	0.98	0.82–1.17	0.88	0.73–1.07
Good health	0.87	0.72–1.06	0.87	0.71–1.06
Fair/Poor health	0.74^d^	0.60–0.92	0.82	0.64–1.05
BMI category				
Healthy weight	Ref	—	Ref	—
Underweight	0.97	0.63–1.50	1.05	0.38–2.95
Overweight	0.99	0.84–1.15	1.05	0.89–1.24
Obese	1.10	0.94–1.29	1.08	0.89–1.30
History of any cancer				
No personal history of cancer	Ref	—	Ref	—
Personal history of cancer	1.36^d^	1.14–1.62	1.30^c^	1.05–1.60

NOTE: Definition of NHIS and data source can be found at https://www.cdc.gov/nchs/nhis/about_nhis.htm.

Abbreviation: Ref, reference category.

^a^Percentage and 95% CI values are weighted.

^b^ *P* < 0.001.

^c^ *P* < 0.05.

^d^ *P* <0.01.

#### Women

##### Predisposing Factors

For each year increase in age past the mean age for screening-eligible women, the odds of colorectal cancer screening increased by 1.06 (*P* < 0.001). The odds of having colorectal cancer screening for married/cohabitating women were 1.41 times the odds for their unmarried counterparts (*P* < 0.001). The odds of having colorectal cancer screening for Black women were 1.51 times the odds for White women (*P* < 0.001). In contrast, the odds of having colorectal cancer screening for AI/AN women were 0.48 times the odds for White women (*P* = 0.036). The odds of having colorectal cancer screening for women with >bachelor's were 1.57 times the odds for women with <HS/GED (*P* = 0.004). The odds of having colorectal cancer screening for foreign-born women residing in the United States for ≤10 years were 0.44 times the odds for women who were born in the United States (*P* = 0.032).

##### Enabling Factors

The odds of having colorectal cancer screening for women at ≤138% FPL were 0.71 times the odds for women at >400% FPL (*P* = 0.007). The odds of having colorectal cancer screening for uninsured women and those with Medicare insurance were 0.43 (*P* < 0.001) and 0.79 (*P* < 0.021) times the odds for women with private insurance. The odds of having colorectal cancer screening for women with a usual source of care in a doctor's office were 2.71 times the odds for women without a usual source of care (*P* < 0.001) and 1.77 times for women with a usual source of care in other types of medical facilities compared with the odds of those without a usual source of care (*P* = 0.008).

##### Need Factors

The odds of having colorectal cancer screening for women with poor/fair health were 0.74 times the odds for women with excellent health (*P* = 0.006). The odds of having colorectal cancer screening for women with a history of cancer diagnosis were 1.36 times the odds for women without a history of cancer diagnosis (*P* = 0.001). See Table 2 and Figures 1 and 2.

#### Men

##### Predisposing Factors

For each year increase in age among men, the odds of colorectal cancer screening receipt increased by 1.07 (*P* < 0.001). The odds of having colorectal cancer screening for married/cohabitating men were 1.40 times the odds of unmarred men (*P* < 0.001). The odds of having colorectal cancer screening for Black men were 1.37 times the odds for White men (*P* = 0.010). The odds of having colorectal cancer screening for men with a HS/GED, some college education, an associate or bachelor's, and >bachelor's degree were greater (ORs: 1.35, 1.50, 1.53, and 1.70, respectively; all *P* < 0.05) than the odds for men with <HS/GED. The odds of having colorectal cancer screening for foreign-born men with ≤10 years of residence in the United States and foreign-born men with >10 years of residence in the United States were 0.25 (*P* = 0.004) and 0.79 (*P* = 0.047) times the odds for men born in the country. The odds of having colorectal cancer screening for men living in the South and Midwest were 0.77 (*P* = 0.005) and 0.73 (*P* = 0.004) times the odds of those living in the Northeast.

##### Enabling Factors

The odds of having colorectal cancer screening for men at ≤138% FPL, at >138%–250% FPL, and at >250%–400% FPL were lesser (ORs:0.59, 0.71, 0.79, respectively; *P* < 0.001, <0.01, <0.05, respectively) than the odds for men at >400% FPL. The odds of having colorectal cancer screening for uninsured men and men with other types of health insurance were lesser than the odds for men with private insurance (OR:0.45, *P* < 0.005; and 0.76, *P* = 0.020). In contrast, the odds of having colorectal cancer screening for men with military insurance was 1.74 times the odds for men with private insurance (*P* = 0.010). The odds of having colorectal cancer screening for men who had a usual source of care at a doctor's office were 4.67 times the odds for men without a usual source of care (*P* < 0.001). For those who had a usual source of care at other medical facilities, their odds of receiving colorectal cancer were 3.48 times the odds of those without a usual source of care (*P* < 0.001).

##### Need Factors

The odds of having colorectal cancer screening for men with a history of cancer diagnosis were 1.30 times the odds for men without a history of cancer diagnosis (*P* = 0.014). See Table 2 and Figures 1 and 2.

## Discussion

Using the Andersen's model as a comprehensive conceptual framework, this study showed receipt of guideline concordant colorectal cancer screenings was associated with several predisposing, enabling, and need factors. While some factors were common for both women and men, other factors were unique to each sex.

Interestingly, our analyses reveal Black respondents exhibited significantly higher odds of colorectal cancer screening receipt compared to White respondents. To the best of our knowledge, this is the first study to show significantly higher odds of colorectal cancer screening among Black adults as compared to White adults in a nationally representative sample. Black adults have historically had lower colorectal cancer screening levels than White adults (17), but this disparity has been decreasing in recent years (17, 18). Our findings suggest that targeted efforts toward modifying enabling factors to increase access (19, 20) and insurance coverage for Black adults (21, 22) may have been successful in improving colorectal cancer screening uptake.

Factors associated with significantly higher odds of colorectal cancer screening uptake for both sexes were: age, being married/cohabitating with a partner, educational attainment higher than a bachelor's degree, having a usual source of care, and having a personal history of cancer. These associations are consistent with the past literature (5, 6, 23–27). As the risk of colorectal cancer increases with age, so does colorectal cancer screening knowledge and awareness (5, 23, 24). Prior research suggests that being married/cohabitating with a partner provides social support and promotes preventive health seeking behaviors (25, 28). Higher educational attainment and engagement in preventive health behaviors have a well-established association (5, 6, 24). We also find that having an established source of care and personal history of cancer increases health care visits which can promote cancer screening uptake (6, 26, 27). Research suggests having insurance coverage leads to having a usual source of care (26, 27) which in turn promotes the use of preventive health care services (21, 29, 30). Conversely, factors correlated with significantly lower odds of colorectal cancer screening uptake in both sexes were: being born outside the United States and living in the United States for ≤10 years, poverty ≤138% FPL, and lack of insurance coverage. These factors are consistent with prior literature, as lack of insurance coverage is often associated with lower cancer screening uptake due to access and financial barriers (6, 23). Being born outside the United States and living in the United States for ≤10 years may pose barriers, including limited access to government health care insurance and other resources in addition to linguistic barriers hindering screening uptake (7).

Among women only, lower odds of colorectal cancer screening was associated with being AI/AN, having Medicare insurance, and being in fair/poor health. Our findings are consistent with prior research which has shown women in fair/poor health may not prioritize colorectal cancer screening above other health conditions (31–33). There is limited research with AI/AN regarding cancer screening behavior, and more research is needed to understand the lower odds of colorectal cancer screening among AI/AN. More research is also needed to understand the disparities for those with Medicare insurance, as having insurance coverage generally improves access by eliminating financial barriers.

Among men only, those with educational attainment greater than HS/GED and having military insurance showed a significantly higher screening uptake. Our findings are consistent with prior research which has shown colorectal cancer screenings have been shown to be high among those with military insurance (34). Factors among men associated with lower screening uptake were being straight/heterosexual, born outside the United States and living in the United States for >10 years, living in the Midwest and South, below 138%–400% FPL, and having other insurance. Our finding that straight men had significantly lower odds of colorectal cancer screening compared to gay or bisexual men is consistent with prior studies (35, 36). One of the reasons for lower screening odds among straight/heterosexual men may be men who have sex with men have higher rates of anal cancers as compared to straight men (37) and, therefore, are more likely to prioritize colorectal cancer screening tests. Foreign-born adults living in the United States for ≤10 years and foreign-born men may face restrictions accessing government health care insurance, language barriers, and other access barriers, regardless of the length of stay in the United States (7). Men living in the South and Midwest had lower odds of receiving colorectal cancer screening, and it is not clear why men from these two U.S. regions are less likely to be screened. Colorectal cancer mortality rates are higher in these regions compared to other U.S. regions (38). Additional research is needed to identify regional cultures or other factors that may affect the willingness of men from these regions to follow colorectal cancer screening recommendations.

Our findings showed there are differences between women and men in the predicting factors that facilitate or constrain colorectal cancer screening. Efforts to improve colorectal cancer screening can be informed by these results, which could be used to guide sex-specific outreach and education interventions. Creating targeted educational materials for women and for men, separately, may generate gains in overall colorectal cancer screenings in the United States. Future research should develop and test colorectal cancer screening information and education based on factors that specifically facilitate or constrain colorectal cancer screenings for women and for men.

### Limitations and Strengths

Survey-based studies are susceptible to some inherent limitations. The data were self-reported and may be subject to recall bias and/or response bias. Moreover, the cross-sectional data did not allow us to examine long-term adherence to colorectal cancer screening and did not report the proportion of participants receiving different screening methods. Nevertheless, this study used a large, nationally representative sample to examine colorectal cancer screening among U.S. women and men. This is the first study to apply Andersen's model to examine how predisposing, enabling, and need domains may affect colorectal cancer screening uptake among women and men separately; in doing so, the study provides a more complete examination of the factors that may influence colorectal cancer screenings by simultaneously examining factors from the model's three domains.

## Conclusion

Encouragingly, this study showed higher odds of colorectal cancer screening receipt among the Black population in a nationally representative sample. However, the overall colorectal cancer screenings among women and men remain lower than national colorectal cancer screening goals, and disparities among certain populations still exist. Continued monitoring of colorectal cancer screenings may help inform and focus efforts toward alleviating colorectal cancer disparities among the most socially and medically vulnerable populations.

## Supplementary Material

Supplementary Table S1 Characteristics of Women and Men Who Were up-to-Date with the USPSTF CRC Screening Guidelines
