# Supplementary material for: Assessment of Colorectal Cancer Screening Disparities in U.S. Men and Women Using a Demographically Representative Sample
Source: Cancer Res Commun. 2022 Jun 30;2(6):561–9. doi: 10.1158/2767-9764.CRC-22-0079 (PMC9645794; doi:10.1158/2767-9764.CRC-22-0079)
Supplement: Supplementary Table S1 — Characteristics of Women and Men Who Were up-to-Date with the USPSTF CRC Screening Guidelines [file crc-22-0079-s01.docx]

**Supplementary Table 1.** Characteristics of Women and Men Who Were up-to-Date with the USPSTF CRC Screening Guidelines

|  |  | **Age-Eligible Women**  Total n=7,503 | **Age-Eligible Men**  Total n=6,486 |
| --- | --- | --- | --- |
| **Variables** | **Operational Definition of Variables** | **N^¶^ (%^¶^, 95%^¶^ CI)**  **or Mean (SE)** | **N^¶^ (%^¶^, 95%^¶^ CI)**  **or Mean (SE)** |
| **Predisposing Factors** | | | |
| Age, in years | Continuous years within age-eligibility range (50-75 years) | 62.9 (0.11) | 62.6 (0.13) |
| **Sexual Orientation** |  | | |
| Straight/heterosexual | Straight, not gay/lesbian | 4,322 (58.4, 56.9 – 59.8) | 3,651 (58.3, 56.8 – 59.7) |
| Homosexual | Homosexual (Gay/lesbian/bisexual, or something else) | 76 (51.7, 41.5 – 61.9) | 104 (71.8, 63.4 – 78.9) |
| **Marital Status** |  | | |
| Married/cohabiting with a partner | Married/living in partnership | 2,444 (60.8, 59.0 – 62.6) | 2,550 (61.7, 60.0 – 63.4) |
| Unmarried | Unmarried /not living in a partnership | 1,999 (53.2, 51.0 – 55.3) | 1,249 (49.1, 46.5 – 51.6) |
| **Race/Ethnicity** |  | | |
| White | Non-Hispanic White | 3,346 (59.8, 58.2 – 61.5) | 3,000 (61.3, 59.7 – 62.9) |
| Black | Non-Hispanic Black | 545 (60.2, 56.6 – 63.8) | 386 (58.3, 53.8 – 62.7) |
| Asian | Non-Hispanic Asian | 174 (51.7, 44.7 – 58.6) | 136 (47.0, 40.3 – 53.9) |
| AI/AN | Non-Hispanic AI/AN | 22 (36.2, 24.0 – 50.5) | 15 (47.3, 33.8 – 61.2) |
| Hispanic | Hispanic | 358 (46.5, 41.9 – 51.2) | 217 (41.8, 36.8 – 46.8) |
| Other race/ethnicity | Non-Hispanic other race | 66 (55.7, 44.6 – 66.2) | 51 (51.8, 40.8 – 62.6) |
| **Educational Attainment** |  | | |
| < HS/GED | Less than a HS graduate or GED (Never attended school/kindergarten only and grade 1-11, 12^th^ grade, no diploma) | 324 (46.8, 41.8 – 51.9) | 277 (41.1, 36.5 – 46.0) |
| HS/GED | A HS graduate or GED or equivalent | 1,076 (53.9, 51.3 – 56.4) | 947 (53.4, 50.6 – 56.2) |
| Some college/no degree | Some college, no degree | 736 (57.8, 54.6 – 61.0) | 601 (60.9, 57.3 – 64.4) |
| Associate-Bachelor’s | An Associate or Bachelor’s Degree (Associate degree: occupational, technical, or vocational program; Associate degree: academic program; and Bachelor’s degree) | 1,650 (61.0, 58.7 – 63.2) | 1,393 (62.9, 60.5 – 65.2) |
| > Bachelor’s | More than a Bachelor’s degree (Master’s degree, Professional School Degree, and Doctoral Degree) | 715 (68.4, 64.9 – 71.7) | 625 (70.6, 67.1 – 73.9) |
| **Nativity** |  | | |
| US citizen by birth | Born in the US | 3,859 (59.7, 58.2 – 61.2) | 3,363 (61.4, 59.9 – 62.9) |
| Living in the US for ≤10 years | Foreign-born, US resident for ≤ 10 years | 18 (24.3, 14.4 – 38.2) | 11 (19.2, 9.3 – 35.4) |
| Living in the US for >10 years | Foreign-born, US resident for ≥ 10 years | 561 (53.0, 49.2 – 56.7) | 421 (46.6, 42.6 – 50.5) |
| **Region of Residence^†^** |  | | |
| Northeast | Northeast (Maine, New Hampshire, Vermont, Massachusetts, Rhode Island, Connecticut, New York, New Jersey, and Pennsylvania) | 799 (58.6, 55.2 – 61.9) | 766 (63.6, 60.2 – 66.9) |
| Midwest | Midwest (Ohio, Michigan, Indiana, Wisconsin, Illinois, Minnesota, Iowa, Missouri, North Dakota, South Dakota, Nebraska, and Kansas) | 1,056 (60.3, 57.3 – 63.2) | 865 (56.7, 53.9 – 59.5) |
| South | South (Delaware, Maryland, Virginia, West Virginia, Kentucky, North Carolina, South Carolina, Tennessee, Georgia, Florida, Alabama, Mississippi, Arkansas, Louisiana, Texas, and Oklahoma) | 1,611 (54.9, 52.8 – 57.0) | 1,367 (55.6, 53.4 – 57.8) |
| West | West (Montana, Idaho, Wyoming, Colorado, New Mexico, Arizona, Utah, Nevada, California, Oregon, Washington, Alaska, and Hawaii) | 1,045 (58.8, 55.5 – 62.0) | 861 (56.7, 53.5 – 59.9) |
| **Area of Residence^‡^** |  | | |
| Large central metropolitan area | Resides in a large central metropolitan area | 1,224 (58.0, 55.4 – 60.6) | 992 (54.2, 51.3 – 57.0) |
| Large fringe metropolitan area | Resides in a large fringe metropolitan area | 1,064 (57.5, 54.7 – 60.1) | 1,028 (62.4, 59.5 – 65.2) |
| Medium-small metropolitan area | Resides in a medium to small metropolitan area | 1,480 (58.7, 56.1 – 61.3) | 1,215 (59.4, 56.9 – 61.8) |
| Non-metropolitan area | Non-metropolitan area | 743 (55.1, 51.4 – 58.7) | 624 (52.9, 49.3 – 56.5) |
| **Enabling Factors** | | | |
| **Employment Status** |  | | |
| Employed | Employed | 2,287 (54.5, 52.7 – 56.3) | 2, 329 (55.2, 53.4 – 57.0) |
| Unemployed | Not employed | 2,224 (61.6, 59.4 – 63.7) | 1,530 (62.7, 60.3 – 65.1) |
| **Problems Paying Medical Bills** |  | | |
| Problems paying medical bills | Yes, problems paying medical bills | 554 (50.4, 46.7 – 54.1) | 373 (48.3, 43.9 – 52.8) |
| No problems paying medical bills | No, no problems paying medical bills | 3,953 (59.2, 57.6 – 60.7) | 3,479 (59.3, 57.8 – 60.8) |
| **Worry about Paying Medical Bills if Sick/in an Accident** | | | |
| Not at all worried | Not at all worried about paying medical bills | 2,616 (63.0, 61.2 – 64.8) | 2,489 (63.4, 61.5 – 65.2) |
| Somewhat worried | Somewhat worried about paying medical bills | 1,283 (56.0, 53.3 – 58.7) | 973 (54.6, 51.9 – 57.3) |
| Very worried | Very worried about paying medical bills | 609 (47.0, 43.6 – 50.5) | 390 (44.2, 40.1 – 48.4) |
| **Federal Poverty Level** |  | | |
| ≤138% FPL | Income less than or equal to 138% of FPL | 654 (44.6, 41.1 – 48.1) | 446 (40.1, 36.1 – 44.1) |
| >138%-250% FPL | Income more than 138% but not more than 250% of FPL | 833 (53.7, 50.4 – 56.9) | 571 (49.4, 45.8 – 53.1) |
| >250%-400% FPL | Income more than 250% but not more than 400% of FPL | 898 (57.9, 54.8 – 60.9) | 743 (56.8, 53.5 – 60.0) |
| >400% FPL | Income more than 400% of FPL | 2,126 (64.5, 62.6 – 66.3) | 2,099 (66.9, 64.9 – 68.8) |
| **Insurance Coverage** |  | | |
| Uninsured | Uninsured | 114 (28.2, 23.3 – 33.7) | 95 (21.2, 17.0 – 26.0) |
| Medicaid | Medicaid insurance | 198 (46.6, 40.4 – 52.9) | 124 (41.8, 34.5 – 49.4) |
| Military insurance | Military insurance | 67 (56.5, 44.9 – 67.4) | 157 (67.7, 59.6 – 74.9) |
| Medicare | Medicare insurance | 923 (61.2, 58.1 – 64.2) | 784 (63.0, 59.5 – 66.3) |
| Private insurance | Private insurance | 2,496 (60.2, 58.3 – 62.0) | 2,150 (61.4, 59.6 – 63.2) |
| Other insurance | Other types of insurance | 706 (62.0, 58.5 – 65.5) | 544 (60.1, 55.7 – 64.4) |
| **Usual Source of Care** |  | | |
| No usual source of care | No usual source of care | 77 (26.9, 21.3 – 33.4) | 94 (18.3, 14.6 – 22.8) |
| Usual source of care – Doctor’s office | A doctor’s office or health center | 4,205 (60.3, 58.9 – 61.8) | 3,308 (62.1, 60.5 – 63.6) |
| Usual source of care – Other medical facility | An urgent care center; a clinic in a drug store or grocery store; a hospital emergency room; a VA Medical Center or VA outpatient clinic; or some other place | 225 (43.6, 38.3 – 49.1) | 452 (54.5, 50.2 – 58.8) |
| **Children in Household** |  | 0.17 (0.01) | 0.19 (0.01) |
| **Need Factors** | | | |
| **Health Status, Self-Reported** |  | | |
| Excellent health | Excellent | 874 (61.5, 58.4 – 64.6) | 718 (61.8, 58.2 – 65.3) |
| Very good health | Very good | 1,587 (61.6, 59.1 – 64.1) | 1,253 (60.9, 58.3 – 63.4) |
| Good health | Good | 1,266 (56.1, 53.5 – 58.6) | 1,171 (55.8, 53.1 – 58.4) |
| Fair/Poor health | Fair or poor | 783 (50.5, 47.4 – 53.5) | 717 (52.5, 49.1 – 55.8) |
| **BMI Category**^§^ |  | | |
| Underweight | Underweight (BMI < 18.5) | 79 (55.4, 45.8 – 64.7) | 23 (60.4, 42.4 – 76.0) |
| Healthy weight | Healthy weight (BMI 18.5 to <25) | 1,419 (59.5, 57.3 – 61.8) | 846 (56.6, 53.4 – 59.7) |
| Overweight | Overweight (BMI ≥ 25 to <30) | 1,280 (57.5, 54.9 – 60.1) | 1,622 (58.7, 56.4 – 60.9) |
| Obese | Obese (BMI ≥ 30) | 1,561 (57.9, 55.4 – 60.3) | 1,352 (58.3, 55.7 – 60.8) |
| **History of Any Cancer** |  | | |
| Personal history of cancer | Yes | 863 (66.2, 63.0 – 69.3) | 677 (71.4, 67.9 – 74.6) |
| No personal history of cancer | No | 3,645 (56.2, 54.7 – 57.8) | 3,180 (55.5, 54.0 – 57.0) |

Note: VA = Veterans Affairs. **^†^**US Census Bureau Regions. ^‡^2013 NCHS Urban-Rural Classification Scheme for Counties. ^§^Category assigned based on BMI calculated by NHIS using study collected height and weight [Weight (kg)/[Height (m) squared]]. ^¶^ Numbers are not weighted; percentages and 95% CIs are weighted.

Data source: 2019 National Health Interview Survey (NHIS). Details of the NHIS can be found at: <https://www.cdc.gov/nchs/nhis/about_nhis.htm>.
